# The behavior and functions of embryonic microglia

**DOI:** 10.1007/s12565-021-00631-w

**Published:** 2021-09-19

**Authors:** Yuki Hattori

**Affiliations:** Department of Anatomy and Cell Biology, Graduate School of Medicine, Nagoya University, 65 Tsurumai, Showa, Nagoya, Aichi 466-8550 Japan

**Keywords:** Cortex, Developing brain, Microglia, Neural progenitors, Neurogenesis

## Abstract

Microglia are the resident immune cells of the central nervous system. Microglial progenitors are generated in the yolk sac during the early embryonic stage. Once microglia enter the brain primordium, these cells colonize the structure through migration and proliferation during brain development. Microglia account for a minor population among the total cells that constitute the developing cortex, but they can associate with many surrounding neural lineage cells by extending their filopodia and through their broad migration capacity. Of note, microglia change their distribution in a stage-dependent manner in the developing brain: microglia are homogenously distributed in the pallium in the early and late embryonic stages, whereas these cells are transiently absent from the cortical plate (CP) from embryonic day (E) 15 to E16 and colonize the ventricular zone (VZ), subventricular zone (SVZ), and intermediate zone (IZ). Previous studies have reported that microglia positioned in the VZ/SVZ/IZ play multiple roles in neural lineage cells, such as regulating neurogenesis, cell survival and neuronal circuit formation. In addition to microglial functions in the zones in which microglia are replenished, these cells indirectly contribute to the proper maturation of post-migratory neurons by exiting the CP during the mid-embryonic stage. Overall, microglial time-dependent distributional changes are necessary to provide particular functions that are required in specific regions. This review summarizes recent advances in the understanding of microglial colonization and multifaceted functions in the developing brain, especially focusing on the embryonic stage, and discuss the molecular mechanisms underlying microglial behaviors.

## Introduction

Microglia, the resident immune cells in the central nervous system (CNS), were first identified and morphologically characterized by Pío del Río Hortega (Río-Hortega 1932). Microglia were initially reported to be innate immune sentinels throughout the CNS, acting as the first line of defense against foreign pathogens and injury-related agents (Streit et al. 1988; Perry et al. 2010; Venegas et al. 2017; Ajami et al. 2018; Picard et al. 2021). It has been assumed that microglia only function in pathological states, but growing evidence has elucidated their essential roles in supporting neuronal differentiation and circuit formation in physiological states (Wake et al. 2009; Paolicelli et al. 2011; Parkhurst et al. 2013; Shemer et al. 2015). Thus, microglia are pivotal players in brain development and the maintenance of homeostasis.

Microglia arise from yolk sac primitive macrophages at embryonic day (E) 8.5 (Alliot et al. 1999; Chan et al. 2007; Ginhoux et al. 2010; Schulz et al. 2012; Kierdorf et al. 2013; Ginhoux and Garel 2018). These cells enter the brain at E9.5, before blood–brain barrier formation, and thereafter colonize the parenchyma through migration and proliferation during brain development (Santos et al. 2008; Hristova et al. 2010; Rigato et al. 2011; Swinnen et al. 2013). Although microglia are a scarce population during the embryonic stage, these cells can extensively survey the brain primordium and associate with surrounding neural lineage cells (Swinnen et al. 2013; Cunningham et al. 2013; Arnò et al. 2014; Hattori and Miyata 2018).

The processes of production, migration and maturation of neural lineage cells are precisely regulated, thereby forming a well-organized neocortex that localizes neural progenitors and neurons based on their differentiation status (Rakic 1974; Nadarajah et al. 2001; Tabata et al. 2012). Most excitatory projection neurons are generated from neural stem cells positioned at the apical side of the neocortical primordium and migrate radially toward the basal surface. On the other hand, most inhibitory interneurons are generated in the ganglionic eminence and migrate tangentially toward the neocortex (Marin et al. 2001; Tamamaki et al. 2003). Importantly, recent studies have revealed that microglia are involved in the regulation of various processes of neuronal production during different stages of development.

This review summarizes the current findings about microglial colonization and their multifaceted functions in the developing brain, specifically focusing on their roles in neural lineage cells during the embryonic stage, and discuss the considerable molecular mechanisms underlying microglial behavior and functions in brain development.

## Entry of microglia into the brain parenchyma

In the developing mouse brain, microglia arise from erythro-myeloid progenitors (EMPs) in the yolk sac at E8.5 (Ginhoux et al. 2010). These cells invade the CNS and colonize the cerebral parenchyma at E9.5 (Alliot et al. 1999; Chan et al. 2007; Ginhoux et al. 2010; Schulz et al. 2012; Swinnen et al. 2013).

Microglial differentiation is regulated by a set of transcriptional programs. The interaction between colony-stimulating factor 1 receptor (CSF1R) and macrophage colony-stimulating factor 1 (CSF-1) is essential for microglial development. In both *Csf1r* knockout and *Csf1* mutant mice, the number of microglia was dramatically reduced (Dai et al. 2002; Ginhoux et al. 2010; Erblich et al. 2011). interleukin (IL)-34, which is another ligand for CSF1R, also contributes to microglial homeostasis (Garceau et al. 2010). In addition, PU.1, which is continuously expressed from EMPs to adulthood microglia, plays an essential role in microglial development. *Pu.1*-deficient mice are devoid of parenchymal microglia in the brain (Beers et al. 2006; Kierdorf et al. 2013). Moreover, interferon regulatory factor 8, which acts downstream of PU.1, has been reported to control the differentiation and functional maintenance of microglia (Kierdorf et al. 2013).

Emerging evidence has suggested several routes of microglial entry into the brain parenchyma. The first way is through blood vessels. An early study reported that microglia colonize the embryonic brain in accordance with vascularization (Earle and Mitrofanis 1998). A study on microglial origins using *Ncx1*^−/−^ mice, in which heartbeat and blood circulation are impaired due to deficiencies in Na^+^/Ca^2+^ exchangers, confirmed that microglial precursors could not invade the brain parenchyma, indicating that these cells enter the embryonic brain in a manner dependent on the establishment of blood circulation (Ginhoux et al. 2010). In addition, the possibility of microglial penetration from the vascular compartment was reported in the developing rat brain (Ashwell 1991) and in avian embryos (Cuadros et al. 1993). The second way is that the ventricle could be a source of microglial progenitors. NADPH oxidase 2 (Nox2), which generates superoxide ions that are released from microglia, regulates microglial chemotaxis, which is mediated by CSF1R and vascular endothelial growth factor receptor-1 (VEGFR1), and promotes the infiltration of these cells from the ventricle into the cortex (Lelli et al. 2013). Such infiltration of microglia from the ventricle has been described along the fourth ventricle of the developing avian brain (Cuadros et al. 1993). The third way is via the meninges. In the early embryonic stage, microglial precursors accumulate near the pial surface. It is possible that these cells traverse the pial surface and infiltrate the parenchyma (Río-Hortega 1932; Boya et al. 1991; Cuadros and Navascués, 2001). Furthermore, although the route of microglial invasion is unknown, a recent study demonstrated that the expression of chemokine C-X-C motif chemokine ligand (CXCL) 12 by Tbr2^+^ intermediate progenitors was involved in microglial recruitment into the pallium (Arnò et al. 2014).

Once microglia are seeded in the parenchyma, these cells proliferate and expand their population until the postnatal stage by reacting to CSF-1, granulocyte macrophage colony-stimulating factor, neurotrophin-3, IL-4 and IL-5 (Navascués et al. 2000). Their cell number peaks by two weeks after birth in rodents, and their density is maintained thereafter by low proliferation levels until adulthood (Dalmau et al. 2003; Ginhoux et al. 2010; Arnoux et al. 2013; Nikodemova et al. 2015).

## Distribution and migration of microglia in the developing cerebral cortex

After microglia enter the brain primordium, they migrate extensively to distinct brain regions in a spatiotemporal pattern throughout the structure. In the early embryonic stage, when the cells of the parenchyma consist of mostly neuroepithelial cells, amoeboid-shaped microglia are observed in the pallium. This morphology is characteristic of immature microglia and has been reported to facilitate their flexible and locomotive migration (Marin-Teva et al. 1998; Monier et al. 2007; Rigato et al. 2011; Swinnen et al. 2013). As development progresses, microglia transform into ramified shapes, and the proportion of cells with long processes increases (Swinnen et al. 2013).

Microglia represent 5–15% of the total cells in the adult brain (Perry et al. 1985), whereas these cells constitute approximately 0.5–1% of the pallial cells in the embryonic brain (Hattori et al. 2020). Of note, microglia in the brain parenchyma change their distribution pattern in a stage-dependent manner. Microglia initially show a homogenous distribution pattern in the pallium in the early embryonic stage, whereas they are temporarily absent from the cortical plate (CP) from E15 to E16 and show preference for colonizing the ventricular zone (VZ), subventricular zone (SVZ), and intermediate zone (IZ). Thereafter, these cells re-enter the CP at E17 (Swinnen et al. 2013; Cunningham et al. 2013; Hattori et al. 2020).

**Fig. 1:**
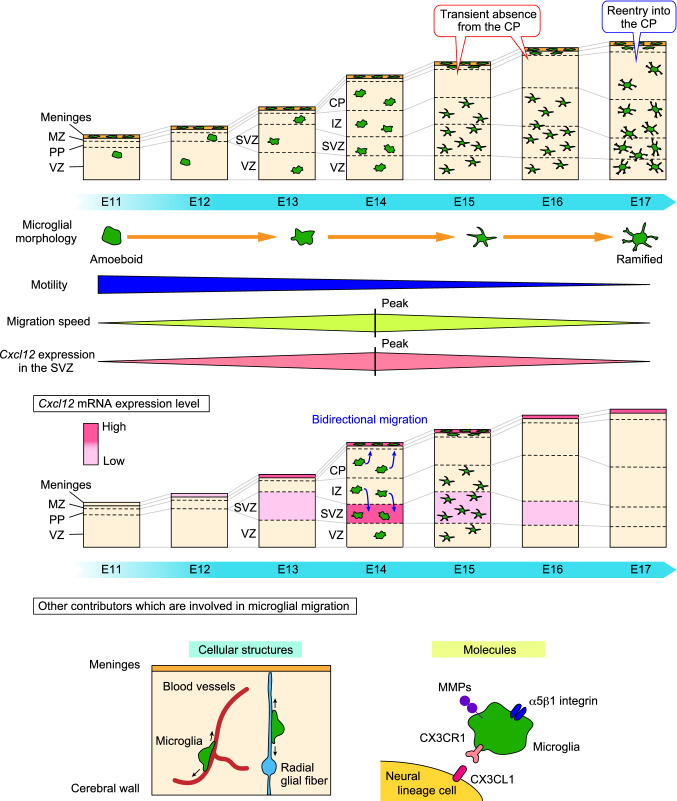
Distribution and migration of microglia in the developing brain. Embryonic microglia change their distribution in a stage-dependent manner in the developing cerebral wall. Microglia are homogenously distributed in the pallium in the early and late embryonic stages, whereas these cells are transiently absent from the CP from E15 to E16. Microglial migration is regulated by multiple factors, i.e., their morphological characteristics, the structures of surrounding cells, such as blood vessels and fibers of radial glia, and some secretory and cell surface molecules. This figure summarizes these factors which have been suggested to contribute to microglial migration in the embryonic stage. The middle panel shows the mRNA expression level of *Cxcl12*, which is produced in the meninges and SVZ. As the *Cxcl12* mRNA expression is most highly expressed in the SVZ at E14, microglia in the IZ might be able to migrate toward the inner region. On the other hand, microglia positioned in the CP are also attracted toward the meninges, thereby accumulating in the MZ. *CP* cortical plate, *IZ* intermediate zone, *MMPs* metalloproteinases, *MZ* marginal zone, *PP* pre-plate, *SVZ* subventricular zone, *VZ* ventricular zone

How is microglial distribution in the developing parenchyma regulated? A growing number of studies have addressed this question, and various scenarios have been suggested thus far. First, blood vessels and blood circulation might regulate microglial migration in the developing brain. Microglial distribution in the brain parenchyma coincides with vascularization, which occurs at E10 in mice (Earle and Mitrofanis 1998). Consistent with this finding, another study reported that microglia reached the area surrounding the spinal cord via the developing vasculature and then proliferated (Rigato et al. 2011). Importantly, vascular sprouts are implicated in microglial migration (Monier et al. 2006). These studies suggest that adhesion molecules or soluble factors derived from vascular endothelial cells might guide microglial migration. Second, radial glial fibers might regulate microglial migration perpendicular to the apicobasal axis. For example, in the quail retina, microglia migrate using radial glial cell fibers (Sanchez-Lopez et al. 2004). Moreover, another study showed that microglia associate with radial cell fibers in the mouse embryonic spinal cord (Rigato et al. 2011). Microglia might use the fibers of radial glia as a scaffold for migration. Third, some soluble molecules and cell surface molecules may support microglial motility. The expression of matrix metalloproteinases (MMPs) 8 and 9, which are known to contribute to extracellular matrix remodeling, contributes to microglial migration and expansion during embryogenesis (Kierdorf et al. 2013). In particular, MMP inhibition impaired microglial spreading. In addition, the fibronectin receptor α5β1 integrin regulates microglial migration in a stage-dependent manner (Smolder et al. 2017). Time-lapse imaging using two-photon microscopy showed that α5β1 regulates microglial migration by promoting migration at E13.5 and by inhibiting migration beginning at E15.5. Recent study reported that microglia migrate along the vasculature by receiving the directional cue of C-X3-C motif ligand 1 (CX3CL1), which is released from surrounding neural lineage cells (Mondo et al. 2020). The CX3CL1/CX3CR1 interaction also helps microglia reach their final destination in the postnatal brain (Paolicelli et al. 2011; Hoshiko et al. 2012). Furthermore, CXCL12 released from the meninges and SVZ regulates microglial bidirectional migration in the mouse embryonic brain (Hattori et al. 2020). As mentioned above, a previous study demonstrated that the expression of the chemokine CXCL12 in intermediate progenitors was involved in microglial recruitment into the VZ/SVZ (Arnò et al. 2014). Hattori et al. further demonstrated that microglia, which were initially positioned in the IZ, migrate toward the SVZ, whereas the cells in the CP move toward the meninges at E14; thus, microglia are expelled from the CP from E15 to E16 (Hattori et al. 2020). Taken together, these findings suggest that the distribution of microglia in the developing cerebral wall is tightly regulated through various mechanisms (Fig. 1).

In addition to these molecular mechanisms, other factors that are known to facilitate microglial migration in the postnatal stage could also contribute to microglial motility in the embryonic stage. First, purinergic receptors have been suggested to be involved in microglial mobility. Purinergic receptor P2X4 and P2Y12 are expressed on microglia and involved in ATP-induced microglial membrane ruffling and migration (Nimmerjahn et al. 2005; Haynes et al. 2006; Ohsawa et al. 2007). Second, evidence suggests a link between microglial distribution and apoptotic cells. A large number of neural lineage cells undergo apoptosis during CNS development, and only selected neural progenitors develop into mature neurons (Oppenheim 1991; Dekkers and Barde 2013; Yamaguchi and Miura 2015; Wong and Marin 2019). Microglia remove apoptotic cells via phagocytosis and therefore regulate the appropriate cell population, ensuring brain homeostasis and neural circuit formation (Ferrer et al. 1990; Egensperger et al. 1996; Sierra et al. 2010; Diaz-Aparicio et al. 2020). A close association between microglial cells and apoptotic neurons has been observed in the neocortex (Upender and Naegele 1999). For example, microglia are attracted by apoptotic cells, leading to developmental neuronal death in some brain areas (Perry et al. 1985; Ashwell, 1990; Marin-Teva et al. 1999). Several “find-me” signals secreted by apoptotic cells, such as the chemokine fractalkine (Truman et al. 2008), the lipid lysophosphatidylcholine (Lauber et al. 2003; Xu et al. 2016), sphingosine 1 phosphate (Gude et al. 2008) and the nucleotides ATP and UTP (Elliott et al. 2009), are involved. Third, some signals from radial glia might recruit microglia. In the early postnatal brain, microglia phagocytically remove the radial fibers of radial glia, which are no longer necessary for radial migration of neural progenitors (Xavier et al. 2015). A more recent paper demonstrated that microglia accumulate around the sub-cerebral projection axons of layer V neurons via the interaction of netrin-G1 and netrin-G ligand to support neuronal survival (Fujita et al. 2020). Further studies are needed to determine whether such molecular mechanisms also contribute to microglial migration and distribution in the developing embryonic brain.

## The roles of microglia in the postnatal stage

In the adult brain, microglia contribute to the maintenance of homeostasis by removing dying neurons or cellular debris and monitoring neuronal circuits for successful synaptic connections in healthy conditions (Paolicelli et al. 2011; Parkhurst et al. 2014; Wake et al. 2019). In pathological contexts, such as neuropsychiatric disorders, neurodegeneration, and infectious diseases, microglia play critical roles as immediate responders with the potential to promote both CNS damage and repair (Venegas et al. 2017; Ajami et al. 2018; Spiller et al. 2018; Hermann and Gunzer 2020).

Accumulating evidence has revealed that microglia also play essential roles in neurogenesis and proper neuronal circuit formation in the developing brain, especially in the postnatal stage. First, microglia have neuroprotective functions. Microglia in the subcortical white matter support the survival of layer V neurons by producing insulin-like growth factor 1 in the postnatal brain (Ueno et al. 2013; Fujita and Yamashita 2021). Microglia also release other factors with neurotrophic functions, such as thrombospondin 1 and 2 (Nagata et al. 1993; Chamak et al. 1995; Christopherson et al. 2005). Thus, microglia support neuronal survival and proper neuronal circuits in the postnatal brain. Second, microglia also regulate the appropriate cell population by removing excess neurons undergoing apoptosis via phagocytosis (Ferrer et al. 1990; Egensperger et al. 1996; Sierra et al. 2010; Dekkers and Barde 2013; Yamaguchi and Miura 2015; Diaz-Aparicio et al. 2020). Third, microglia are involved in synaptic pruning and neuronal turnover (Paolicelli et al. 2011; Miyamoto et al. 2016). Fourth, recent studies have suggested a potential role of microglia in gliogenesis. An in vitro study showed that microglia promoted the differentiation of astrocytes through the release of IL-6 (Nakanishi et al. 2007). Another study revealed that microglia released cytokines, such as IL-1β, IL-6, tumor necrosis factor alpha (TNF-α), and platelet-derived growth factor (PDGF), that modulate the survival and maturation of oligodendrocytes (Shigemoto-Mogami et al. 2014). Sherafat et al. reported that microglial neuropilin 1 promotes oligodendrocyte progenitor cell (OPC) expansion during development and remyelination (Sherafat et al. 2021).

## The roles of microglia in the embryonic stage

In contrast, research on the functions of embryonic microglia is lacking. However, recent progress suggests multifaceted functions of these cells in this stage.

### Promoting the differentiation of neural progenitors

Microglia preferentially colonize the VZ/SVZ, which is the neurogenic region of the telencephalon in rodents and primates (Antony et al. 2011; Cunningham et al. 2013; Arnò et al. 2014; Squarzoni et al. 2014). Microglia have been reported to play essential roles in regulating the number of neural progenitors in the VZ/SVZ. Arnò et al. showed that the number of Tbr2^+^ cells in the SVZ decreased slightly at E13.5 and more dramatically at E17.5 in *Csf1r*^*flox/flox*^ mice, in which microglia were conditionally depleted, indicating that microglia increase the number of Tbr2^+^ cells (Arnò et al. 2014). Consistent with this finding, Hattori and Miyata reported that in vivo pulse-chase experiments combined with the elimination of microglia using clodronate liposomes or the arrest of microglial motility by C-X-C chemokine receptor 4 (CXCR4) antagonist administration led to a prominent decrease in Tbr2^+^ intermediate progenitors in the SVZ and an increase in Pax6^+^ neural stem cells (Hattori and Miyata 2018). In addition, in vitro coculture of microglia and neural progenitors showed that the proportion of Tbr2^+^ cells was increased, whereas that of Pax6^+^ cells was decreased. These findings suggest that microglia promoted the differentiation of neural stem cells into intermediate progenitors. However, the molecular mechanism remains to be elucidated. Since previous reports showed that microglia secrete some cytokines, such as IL-1β, IL-6, and TNF-α, all of which increase neuronal differentiation in the early postnatal stage (Giulian et al. 1986; Nakanishi et al. 2007; Shigemoto-Mogami et al. 2014), it is possible that some of these factors also contribute to neurogenesis in the embryonic stage.

### Regulation of the number of neural progenitors by phagocytosis

On the other hand, microglia also regulate the population of neural progenitors through phagocytosis. Microglia contribute to brain development by digesting dead cells. Hamilton et al. showed that the number of developmental apoptotic cells was increased in ribonuclease T2-deficient zebrafish larvae due to a defect in apoptotic cell clearance by microglia (Hamilton et al. 2020). In addition, microglia phagocytose live neural progenitors. Cunningham et al. demonstrated that microglia phagocytically reduced the number of Tbr2^+^ neural progenitors in the VZ/SVZ of the developing rat brain (Cunningham et al. 2013). The authors reported that the induction of proinflammatory (M1) microglia using lipopolysaccharide decreased the abundance of Pax6^+^ and Tbr2^+^ cells during the late embryonic period, whereas inactivation by minocycline, the induction of an anti-inflammatory (M2) phenotype by doxycycline and the removal of microglia by liposomal clodronate increased the abundance of both Pax6^+^ and Tbr2^+^ cells in the late embryonic and early postnatal SVZ/VZ.

The reason is unclear why the results regarding the effect of microglial loss on the number of Tbr2^+^ cells were contradictory (Cunningham et al. 2013; Arnò et al. 2014; Hattori and Miyata 2018). One possibility is that there might be a difference in the reactivity of neural progenitors to microglia among animal species. Studies that showed that microglial loss led to a decrease in Tbr2^+^ cells were conducted in mice, whereas an increase in Tbr2^+^ cells when microglia were depleted was observed in rats. Second, there might be differences in the experimental time schedules. Hattori and Miyata performed fate tracking analysis of daughter cells generated from 5-bromo-2’-deoxyuridine (BrdU)-labeled progenitors 24 h after the manipulation. In this context, the period of cell fate choice was mainly analyzed, rather than the subsequent cell survival phase. During a longer time of analysis, secondary effects might be detected. Overall, microglia play dual roles, promoting the differentiation of neural progenitors and regulating the number of neural progenitors to acquire an appropriate cell population.

### Wiring of interneurons

Microglia are also involved in the wiring of interneurons. Interneurons, which originate in the ganglionic eminence, migrate tangentially and enter the pallium (Marin et al. 2001; Tamamaki et al. 2003; Lim et al. 2018). Microglia have been recently reported to be involved in the entrance and positioning of interneurons. Squarzoni et al. demonstrated that microglial depletion and maternal immune activation had a marked impact on the positioning of Lhx6^+^ interneurons (Squarzoni et al. 2014). Lhx6-expressing interneuron is locally restricted in layer V of the neocortex under physiological conditions, whereas this interneuron subpopulation entered prematurely into the neocortex and had a less focal distribution around layer V. Moreover, they showed that microglial fine-tuning activity for neocortical interneuron positioning is regulated by CX3CR1 and DNAX-activating protein of 12 kDa (DAP12) signaling.

### Gliogenesis

A more recent paper demonstrated that microglia influence nearby glial precursors through cytokine signaling and support appropriate oligodendrocyte maturation in the developing tuberal hypothalamus in the embryonic stage (Marsters et al. 2020). The authors reported that C–C motif chemokine ligand 2 (CCL2) and CXCL10 released from microglia affected neuronal differentiation and promoted oligodendrocyte production at the expense of astrocyte differentiation.

Taken together, these findings suggest that microglia play various roles and are involved in neurogenesis, gliogenesis, the regulation of the number of neural progenitors, and neuronal circuit formation in the embryonic stage.

## The mechanism of transient microglial absence from the mid-embryonic CP

As noted previously, Hattori et al. reported that microglia bidirectionally migrate throughout the developing mouse brain (Hattori et al. 2020). Time-lapse imaging of microglia in cerebral wall slices prepared from CX3CR1-GFP mice, in which microglia were labeled with GFP, revealed that most microglia that were initially localized in the CP tended to migrate toward the basal lamina and accumulate in the marginal zone. Of note, when the meninges were removed, these cells could not translocate their soma in a basal direction. On the other hand, microglia that were initially present in the IZ tended to move in an apical direction. Based on such migratory characteristics of microglia, it was hypothesized that molecules specifically expressed in both the meninges and the inner region of the cerebral wall could attract microglia. CXCL12 was identified as a candidate molecule because its mRNA was specifically expressed in the meninges and SVZ, as detected by in situ hybridization. *Cxcl12* mRNA expression was sustained in the meninges from E12 to the later stage, and the highest expression level was in the SVZ at E14. To test the hypothesis that the CXCL12/CXCR4 interaction is involved in bidirectional microglial migration, time-lapse imaging was performed on microglia in *Cxcr4*^−/−^ mice crossed with CX3CR1-GFP mice. In *Cxcr4*^−/−^ mice, microglia showed reduced migration at E14 and manifested an abnormal distribution pattern at E15; a significant increase in microglia positioned in the CP was observed compared to that in wild-type mice. These results suggest that the CXCL12/CXCR4 system plays an essential role in the attraction of microglia and their proper positioning in the mid-embryonic cerebral wall (Fig. 1).

However, the possibility that other molecular mechanisms collaboratively regulate the transient absence of microglia from the mid-embryonic CP is not excluded. This is because it was observed that some microglia could still exit the CP of the cerebral walls at E14 in *Cxcr4*^*−/−*^ mice. The reason was that post-migratory neurons positioned in the CP might express or release molecules that divert microglia from the CP. For example, the chemo-repulsive molecule Slit1 is highly expressed in the embryonic CP (Whitford et al. 2002). Slit1 might regulate microglial expulsion via recognition by PlexinA1 expressed on microglia (Delloye-Bourgeois et al. 2015). In addition, the migration of OPCs in the embryonic optic nerve is regulated by a balance of effects mediated by semaphorins and netrins (Spassky et al. 2002). Similar to this case, microglial migration could be guided by a gradient of cues, such as semaphorins and netrins.

Moreover, the molecular mechanism underlying microglial entry into the CP at E17 remains elusive. *Cxcl12* mRNA expression in the SVZ was highest at E14 and then declined toward the late embryonic stage, but its expression in the meninges was sustained throughout the embryonic stage. It is unclear why microglia can reenter the CP, although the meninges highly express CXCL12 in the late embryonic stage. Thus, three possibilities can be considered. First, the reactivity of microglia to CXCL12 might change. Since CXCR4 expression in microglia persists until the perinatal period (Thion et al. 2018), intracellular processing of CXCL12 signaling might be negatively regulated toward the end of the embryonic stage. Second, microglia might lose their migration capacity. As mentioned above, microglia change their morphology and transform into ramified cells toward the late embryonic stage (Swinnen et al. 2013). Such morphological changes might affect microglial migration. Third, newly formed vasculature that invades perpendicularly in the CP against the apical surface might guide microglial infiltration. Previous studies have shown that neovascularization in the CP proceeds in the late embryonic stage (Puelles et al. 2019). Further studies are needed to elucidate the mechanism of microglial reentry into late embryonic CP.

In summary, the localization of microglia in the cortical wall is spatiotemporally regulated via complicated mechanisms involving multiple factors, including the CXCL12/CXCR4 system, throughout the developmental stage.

## The significance of transient microglial absence

Nervous system development proceeds through the ordered generation of various cell types, which are initially produced from neural stem cells of the same origin (Rakic 1972, 1974; Nadarajah et al. 2001; Valiente and Marin 2010). The fate of each cell type is determined in a step-by-step manner through the sequential expression of temporal transcription factors, which are critical for guiding neurons toward their final state (Telley et al. 2016). Once early-born neurons initially form the CP, late-born neurons pass through these cells and accumulate in the CP; therefore, the CP is composed in an inside-out pattern (Lodato et al. 2015). Following birth-dependent neuronal specification that occurs in the VZ/SVZ (Arlotta et al. 2005; Molyneaux et al. 2007), recent studies indicate that post-migratory neurons undergo subsequent differentiation to confer their projection subtype identity in the CP as a final step (Kwan et al. 2008; Lai et al. 2008; Oishi and Nakajima 2018). For example, Sox5 regulates the differentiation of post-migratory early-born sub-plate and deep layer neurons (Kwan et al. 2008; Lai et al. 2008). Another study reported that the knockdown of protocadherin (Pcdh20) caused the mal-positioning of future layer 4 neurons in layer 2/3 and induced these cells to acquire layer 2/3 characteristics (Oishi et al. 2016). Hattori et al. recently reported that artificial exposure of microglia to post-migratory neurons in the CP resulted in disturbances in the expression patterns of neuronal subtype-associated genes, which are essential for proper neuronal differentiation (Hattori et al. 2020). Post-migratory neurons that are exposed to excessive numbers of microglia fail to appropriately express subtype-associated transcription factors, showing a tendency for reduced expression of deep layer marker genes, such as Ctip2, and increased expression of typical upper layer marker genes, such as Satb2 and Ctip2. This result indicates that transient absence of microglia from the mid-embryonic CP is critical for proper differentiation of post-migratory neurons.

On the other hand, interneurons have been shown to undergo modifications via environmental factors to acquire their final interneuron subtypes. A majority of cortical GABAergic interneurons are classified as either parvalbumin (PV)-positive interneurons or somatostatin (SST)-positive interneurons (Kawaguchi and Kubata 1997). Of note, Tanaka et al. showed that the proportions of PV-positive interneurons and SST-positive interneurons differentiated from medial ganglionic eminence (MGE) cells transplanted into the medial prefrontal cortex were different from those differentiated from MGE cells transplanted into the occipital cortex, suggesting that environmental factors affect final interneuron subtypes (Tanaka et al. 2011). Another transplantation study reported that the host environment regulates the proportion of interneuron subtypes in the brain region (Quattrocolo et al. 2017). Taken together, these findings suggest that the cortical neuronal network is largely permissive, and the progressive maturation of neurons in their environment requires the orchestration of gene and protein expression that is partly guided by external cues.

**Fig. 2:**
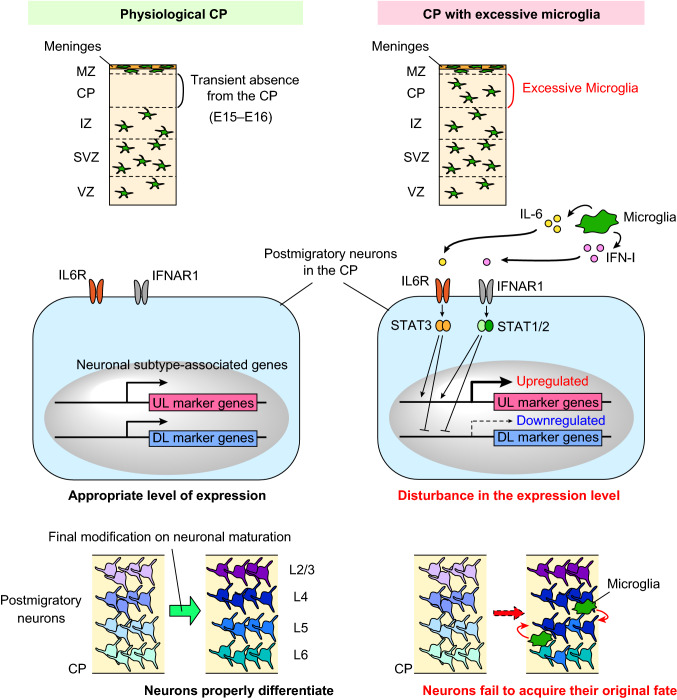
Excessive microglia disturb the differentiation and maturation of post-migratory neurons in the CP. Microglia transiently exit the CP from E15 to E16. If microglia are inadvertently positioned in the CP, these cells would disturb the expression properties of subtype-associated transcription factors in post-migratory neurons by inducing a reduction of the expression of deep layer (DL) marker genes and an increase of typical upper layer (UL) marker genes. Moreover, IL-6 and IFN-I released from microglia have been identified as two important mediators which participate in the destabilization of the expression of neuronal subtype-associated genes. Thus, the developing cortex might expel microglia from the mid-embryonic CP to appropriately fine-tune the expression of molecules needed for proper differentiation of post-migratory neurons to secure the establishment of functional cortical circuit. *CP* cortical plate, *IFNAR1* interferon alpha/beta receptor, *IFN-I* type I interferon, *IL-6* interleukin-6, *IL6R* interleukin 6 receptor, *IZ* intermediate zone, *L* layer, *MZ* marginal zone, *SVZ* subventricular zone, *VZ* ventricular zone

Hattori et al. showed that IL-6 and type I interferon (IFN-I) are primary mediators that disturb the expression of essential genes for proper neuronal maturation (Hattori et al. 2020) (Fig. 2). However, the detailed mechanism by which these cytokines modulate the expression of transcription factors that are fundamental for neuronal differentiation remains unclear. It is possible that multiple factors are involved in this transcriptional change. IL-6, when recognized by the IL-6 receptor (IL6R), activates signal transducer and activator of transcription (STAT) 3 via tyrosine kinase 2 (TYK2) and Janus kinase (JAK) 2-mediated phosphorylation, thereby inducing the transcriptional activation of target genes (Morris et al. 2018). Of note, a previous study reported that the expression of Satb2 was upregulated cell extrinsically by ciliary neurotrophic factor and leukemia inhibitory factor, which are members of the gp130 cytokine family, in cervical ganglion neurons undergoing neurotransmitter transspecification (Apostolova et al. 2010), suggesting that IL-6, which converges on the gp130 pathway, may similarly enhance the transcription of Satb2 in post-migratory neurons. Moreover, other studies demonstrated that IL-6 upregulates DNA methyltransferase 1 (DNMT1) and downregulates ten–eleven translocation 3 (TET3) via the JAK2-STAT3 signaling pathway, causing the DNA methylation of NeuroD1 and resulting in the switch from neurogenesis to astrogliogenesis (Fan et al. 2005; Kong et al. 2019). On the other hand, IFN-I, which is recognized by interferon alpha/beta receptor (IFNAR1), activates STAT1 and STAT2 via TYK2 and JAK1 and thereafter induces the expression of IFN-stimulated genes (McNab et al. 2015). IFN-I epigenetically regulates the functions of a wide variety of cell types, including macrophages (Stefan-Lifshitz et al. 2019) and pancreatic β cells (Kroetz et al. 2015), thereby contributing to the initiation of diseases. Therefore, such regulatory mechanisms mediated by IFN-I and IL-6 could cause the posttranscriptional modification of transcription factors that are essential for the differentiation of post-migratory neurons. Thus, microglia, if they are inadvertently localized in the mid-embryonic CP, would disturb the stabilization of the molecular properties of post-migratory neurons via the release of IL-6 and IFN-I. The developing cortex expels microglia from the mid-embryonic CP to appropriately fine-tune the expression of transcription factors that are essential for the proper differentiation of post-migratory neurons, thus securing the establishment of a functional cortical circuit.

## Conclusion

In the developing brain, microglial colonization in the brain parenchyma and distribution are tightly regulated in a spatiotemporal manner by a vast series of molecular mechanisms. Microglia might be exposed to many different cues from other CNS cells via direct cell–cell contact or react to extrinsic soluble factors, which enable microglia to migrate at a proper timing. However, it should be highlighted that many previous studies on microglial colonization and distribution have been based on immunohistochemical analyses at specific time points. Research on microglial behavior using time-lapse imaging, such as slice culture-based live observation or in vivo monitoring, will certainly enable us to overcome the limitations of techniques such as immunohistochemistry and contribute to a better understanding of the dynamics of microglial colonization.

Microglia play diverse and essential roles in various developmental processes at different stages of development, such as neurogenesis, axonal guidance, apoptotic cell phagocytosis and synapse remodeling. In this review, the roles of microglia in neural lineage cells have been focused, but emerging evidence has shown that microglia interact with vasculature. In the pathological state, microglia are rapidly attracted to the vasculature following breakdown of the BBB (Barkauskas et al. 2015; Dudvarski Stankovic et al. 2016). Microglia also react to the inflammation and then disrupt the BBB integrity, thereby permeabilizing the BBB barrier (Dudvarski Stankovic et al. 2016; Zhao et al. 2018; Haruwaka et al. 2019). In the healthy brain, microglia regulate vascular formation and complexity in the developing brain and retina (Checchin et al. 2006; Fantin et al. 2010; Rymo et al. 2011; Dudiki et al. 2020). In addition, microglia use the blood vessels to migrate throughout the CNS structure (Grossmann et al. 2002; Checchin et al. 2006; Monier et al. 2007; Fantin et al. 2010; Mondo et al. 2020). Thus, microglial functional failure at critical time points during brain development might have detrimental effects on such events and cause neurological diseases. An understanding of microglial development and function is needed and contributes to the establishment of new treatments or solutions for such disease development.

A recent study demonstrated that microglial gene expression profiles change in a stage-dependent manner, suggesting that microglia are heterogenic and have distinct characteristic roles at each developing stage (Matcovitch-Natan et al. 2016; Hammond et al. 2019; Kracht et al. 2020). In addition to such genetic regulation, local factors also modulate the regional heterogeneity of microglia. The possibility of the involvement of local factors in microglial characterization in the adult brain has been suggested (Grabert et al. 2016; Hart et al. 2012; Ribeiro Xavier et al. 2015; Schnell et al. 1999). Of note, environmental factors also change microglial phenotype during development. The most striking example is probably the influence of the microbiota on microglial maturation. A recent study showed that the morphology and expression of genes that are critical for maturation in microglia are altered in germ-free mice, suggesting that the host microbiota controls microglial phenotype throughout life (Erny et al. 2015). Furthermore, another study revealed that the maternal microbiota affects embryonic microglia, as shown by major transcriptional differences observed between germ-free and control embryos (Thion and Garel 2017; Thion et al. 2018). This study raised the possibility that the gut microbiota has an impact on brain circuit formation and neurological disease manifestation through microglial dysfunction. Notably, maternal inflammation, such as viral or bacterial infections, has been reported to cause diseases, such as autism spectrum disorders and epilepsy (Knuesel et al. 2014). Furthermore, maternal inflammation causes abnormal microglial distribution and motility in the embryonic stage (Squarzoni et al. 2014; Ozaki et al. 2020). Maternal cold stress affects microglial effects on nearby neural progenitors in a sexually dimorphic manner in the embryonic hypothalamus (Rosin et al. 2021). Taken together, these findings suggest that microglial characterization is determined through both an intrinsic transcriptional program and extrinsic environmental factors. Further studies are needed to reveal how such factors affect microglial development, colonization, and function.

It is certainly worth investigating whether such abnormal behaviors of microglia following early dysfunction might lead to various neurological disorders. Fully understanding microglial regulation and functions will not only contribute to a better understanding of normal CNS development but also establish effective therapies to alleviate these disease symptoms.
